# Inner experience in the scanner: can high fidelity apprehensions of inner experience be integrated with fMRI?

**DOI:** 10.3389/fpsyg.2014.01393

**Published:** 2014-12-09

**Authors:** Simone Kühn, Charles Fernyhough, Benjamin Alderson-Day, Russell T. Hurlburt

**Affiliations:** ^1^Center for Lifespan Psychology, Max Planck Institute for Human DevelopmentBerlin, Germany; ^2^Department of Psychology, Durham UniversityDurham, UK; ^3^Department of Psychology, University of NevadaLas Vegas, Las Vegas, NV, USA

**Keywords:** inner speech, inner speaking, inner hearing, inner experience, introspection, fMRI, descriptive experience sampling, mind wandering

## Abstract

To provide full accounts of human experience and behavior, research in cognitive neuroscience must be linked to inner experience, but introspective reports of inner experience have often been found to be unreliable. The present case study aimed at providing proof of principle that introspection using one method, descriptive experience sampling (DES), can be reliably integrated with fMRI. A participant was trained in the DES method, followed by nine sessions of sampling within an MRI scanner. During moments where the DES interview revealed ongoing inner speaking, fMRI data reliably showed activation in classic speech processing areas including left inferior frontal gyrus. Further, the fMRI data validated the participant’s DES observations of the experiential distinction between inner speaking and innerly hearing her own voice. These results highlight the precision and validity of the DES method as a technique of exploring inner experience and the utility of combining such methods with fMRI.

## INTRODUCTION

There is a large literature in both cognitive neuroscience and behavioral psychology that seeks to characterize aspects of inner experience. A growing subset of studies has sought to use fMRI to identify neural correlates of inner experience, for example by studying thinking that is not tightly related to the current task. This has been variously called mind wandering (Kane et al., 2007; Killingsworth and Gilbert, 2010; Smallwood, 2013); inner experience during resting state (Fell, 2013); undirected thought (Klinger and Cox, 1995); stimulus independent thought (Gilbert et al., 2007; Mason et al., 2007); task unrelated thought (Giambra, 1989; Smallwood, 2013); spontaneous thought (Klinger, 2009); daydreaming (Singer, 1966); free thought (Doucet et al., 2012); and so on. Those terms have somewhat different meanings, but all depend centrally on directly apprehended phenomena of inner experience: for example, at time *t* I was thinking about my girlfriend while I was supposed to be doing my homework. Following Christoff (2012), we will refer to all of these as “undirected thoughts.”

Capturing the incidence and flow of such inner experience is an intriguing challenge for neuroimaging. Undirected thoughts have been mostly studied either by contrasting passive “baseline” periods with an active, stimulus-driven task (see Shulman et al., 1997) or in terms of connectivity between different brain regions in a resting state (e.g., Greicius et al., 2008). The identification of brain areas and networks associated with rest allows for brain-behavior correlations to be examined: for instance, self-reports of a particular experience, such as anxiety, can be correlated across a group of individuals with responses in particular brain regions (McGuire et al., 1997; Dennis et al., 2011; Doucet et al., 2012). Of particular note, the “default mode network” (DMN) refers to a set of regions that appear to be consistently anti-correlated with task-positive activity and associated with introspective processes (Raichle and Snyder, 2007; Buckner et al., 2008). There are, however, a range of other networks also thought to be active during resting periods, such as those guiding attentional and executive control (Smallwood, 2013).

Recently a number of novel methods have been developed to induce and examine undirected thoughts, most of which have targeted instances of mind wandering. One approach is to train participants on a particular cognitive task that is conducive to mind wandering, deploy the task in the scanner, ask participants to report instances of mind wandering, and then correlate this with DMN activity (McKiernan et al., 2006; Mason et al., 2007). Another approach has been to ask participants to respond to random probes and report on their subjective state while performing a repetitive task in the scanner (Christoff et al., 2009; Andrews-Hanna et al., 2010; Stawarczyk et al., 2011) or during task-free rest periods (Tusche et al., 2014). For instance, Christoff et al. (2009) probed participants during a go/no-go task and asked them to use Likert scales to indicate (a) whether their attention was focused on the task and (b) whether they were aware of their attentional state at that time (“concurrent awareness”). Self-reported mind wandering states were associated with increased activation of typical areas of the DMN and the central executive network, and this was particularly the case for mind wandering without concurrent awareness (Christoff et al., 2009).

These methods offer novel ways of investigating undirected thoughts. Nevertheless, they all involve a trade-off of some kind. Experiences can be sampled immediately by asking participants to make a forced-choice discrimination while in the scanner. But such techniques almost always preclude detailed descriptions of experience; experience sampling techniques are typically used simply as classifiers of two or three different “modes” of thinking (such as being “on-task” vs. “off-task”; Christoff et al., 2009). If researchers have wanted to know more about the nature of that state, they have occasionally inquired via questionnaire or interview, but these inquiries have always been retrospective, outside the scanner (Delamillieure et al., 2010; Doucet et al., 2012; Diaz et al., 2013). However, such retrospective reporting undermines the immediacy of the sampling method, because self-reporting on experience after a delay may be subject to a range of reporting biases (Fell, 2013) and is likely to reflect presuppositions about the experience rather than direct apprehensions of experiential phenomena (Hurlburt, 2011).

A technique with the potential to offer an alternative method is descriptive experience sampling (DES; Hurlburt, 1993, 1997, 2011; Hurlburt and Heavey, 2001, 2002, 2006; Hurlburt and Akhter, 2006; Hurlburt and Schwitzgebel, 2007; Heavey and Hurlburt, 2008; Heavey et al., 2012; Hurlburt et al., 2013). DES uses a random beeper to signal participants to attend to the experience that is ongoing at the moment of the beep, and immediately thereafter to jot down notes about that ongoing experience. Within 24 h the DES investigator conducts an “expositional interview” to discover the characteristics of the beeped pristine experience. This process is repeated over a number of days (usually 4–6). DES differs from other experience sampling methods in that it aims to help participants bracket their tendencies to report presuppositions and thereby aims to produce high fidelity descriptions of “pristine” inner experiences—thoughts, feelings, sensations, seeings, hearings, and so on as they naturally occur in a person’s everyday environment. It therefore does not specify characteristics that are to be rated in a forced-choice manner, but instead explores characteristics that emerge (Hurlburt, 2009) from the individual person’s own experience, untainted (as much as possible) by the investigator’s predilections.

There are five experiential phenomena that DES claims occur frequently (Heavey and Hurlburt, 2008); we will call those the 5FP for “5 frequent phenomena”: inner speaking (Hurlburt et al., 2013), inner seeing (aka visual imagery), unsymbolized thinking (Hurlburt and Akhter, 2008), sensory awareness (Hurlburt et al., 2009), and feelings (Heavey et al., 2012). However, DES is fundamentally an idiographic procedure, which implies that in any particular individual it is possible that one or more of those characteristics are present, that none of those characteristics are present, or that one or another of those characteristic might be present in an idiosyncratic way. Thus the 5FP are sometimes convenient ways to characterize an individual, sometimes not. That is, DES in general has no expectations about what might emerge as the characteristics of any individual’s inner experience. The DES procedure is “open-beginninged” (Hurlburt and Heavey, 2006; Hurlburt and Schwitzgebel, 2007; Hurlburt, 2011) in the sense that the DES interviewer does *not* set out to inquire whether a participant is innerly speaking, or is innerly seeing, or so on. If a participant, describing her experience without benefit of the DES categories, describes (for example) inner speaking (regardless of the words she chooses in so describing), the DES investigator will continue to explore whether inner speaking was ongoing at the moment of the beep.

The DES process can produce surprising results. For example, inner speech is held by some to occur during every waking moment (Archer, 2003; Baars, 2003; Ihde, 2012) and by others to occur about three-quarters of the time (e.g., Klinger and Cox, 1995). DES studies by Hurlburt and colleagues, however, hold that inner speaking is present on average only about one-quarter of the time and in many people never or only very rarely (Heavey and Hurlburt, 2008; Hurlburt et al., 2013). Hurlburt (2011) has argued that discrepancies in reports of many experiential phenomena are common because many if not most people do not know the characteristics of their own inner experience, and instead rely on presuppositions that interfere with their ability to report reliably and accurately. Furthermore, Heavey and Hurlburt (2008) report huge individual differences in the frequency of inner seeing, feelings, and other forms of inner experience, raising further concerns about how assumptions about the nature of inner experience might influence self-report.

In contrast to other methods, then, DES aims to clear out these assumptions and train participants to be more careful in reporting their experiences by avoiding generalizations, focusing on a precise moment (just before the beep), and interviewing participants not just once but on multiple occasions, in an iterative manner (Hurlburt, 2009, 2011). It remains to be seen whether such an idiographic procedure can be of value to neuroscientific investigations of inner experience and undirected thought. The challenge is to convert such a highly detailed method for use with fMRI, and then to test (using evidence from cognitive neuroscience) the claim that DES can offer a reliable reflection of participants’ experiences.

Two of us (SK, a neuroscientist, and CF, a psychologist) invited DES-creator Hurlburt (hereafter called RH, whose work we knew but with whom we had no relationship) to put the DES method to the test. We recruited five participants, all unknown to RH and unrelated to us, and invited RH to perform a typical DES investigation with each. Data on all five participants is reported elsewhere as part of a wider study (Hurlburt et al., in preparation). Our aim here is to establish proof-of-principle in a single participant that idiographic investigation of inner experience, such as is provided by DES, can be successfully combined with fMRI. As such we only report here on one of those participants, “Lara,” an 18 year-old-woman. As with the other participants, RH trained Lara in the usual DES way (four sampling days in her natural environments); then we delivered random DES beeps to her in nine sessions while she was in an MRI scanner, four random beeps per 25-min session. Immediately after each session, RH conducted a typical DES interview with her about the four beeped experiences. Before the fMRI data were analyzed, RH descriptively characterized those 36 in-the-scanner moments of Lara’s experience; these 36 DES characterizations were used to form participant-specific categories on which the fMRI contrasts would be based.

In a preliminary phase at the start of the study, for possible comparison with the DES results, we also asked Lara to perform conventional neuroscience imagination tasks in the scanner: we directed her to generate specific verbal, auditory, visual, emotional, and somatosensory imagery when instructed by prompts such as “to see a pencil” or “to say ‘lamp’.” These conventional tasks could then be used to test whether the neuroimaging results derived from the DES method were plausible, localizable, and comparable to brain activity determined by conventional (non-introspective) means.

Because of the idiographic, open-beginninged nature of DES, at the outset of Lara’s DES sampling, we had no expectations about whether one or more of the 5FP might emerge as a salient characteristic of her inner experience. Nonetheless, to facilitate nomothetic comparison it is useful to consider the 5FP if they emerge. It turned out that for Lara, according to DES, sensory awareness was the most frequent of the 5FP (27 occasions or 75% of Lara’s 36 in-scanner samples); however, its varying modality (visual, bodily, auditory, etc.) made it an unlikely candidate for neural correlation. Inner seeing occurred in 8 (22%) of Lara’s 36 in-scanner samples; however, inner seeing had never occurred in Lara’s natural environment DES sampling, so it seemed likely to be an artifact of the scanner situation. Inner speaking occurred in 8 (22%) of Lara’s 36 in-scanner samples; it had also occurred in 13% of Lara’s natural environment DES sampling, so inner speaking seems a good candidate for further consideration. Of the remaining 5FP phenomena, unsymbolized thinking and feelings were too rare [one occasion (3%) each]. (Percentages do not add to 100% because multiple ratings are possible.) Thus, as a result of Lara’s idiographic experiential result, we will focus here on Lara’s inner speech-related neural processing.

The neuroimaging literature suggests that language or speech-based samples are typically associated with brain areas such as left inferior frontal gyrus (IFG), superior temporal sulcus (STS), and the superior and middle temporal gyri (Price, 2012; Gernsbacher and Kaschak, 2003), with inner speech processes in particular being linked to activation in left IFG and lateral temporal areas (Shergill et al., 2001, 2002; Jones and Fernyhough, 2007; Marvel and Desmond, 2012; Kühn et al., 2013). Damage to left IFG is also associated with impairment on inner speech tasks (e.g., Geva et al., 2011). If Hurlburt’s claims about DES were correct, the fMRI data collected during Lara’s eight inner-speaking (according to DES) moments should involve brain activation in some or all of the above-mentioned areas. If this was observed, it would provide support to the principle that DES and fMRI might be profitably combined.

## MATERIALS AND METHODS

The study was conducted according to the Declaration of Helsinki, with approval of the German Psychological Society Ethics Committee.

Lara was scheduled for 19 sessions across a 2-week period, which was divided into four phases. She was right-handed and aged 18.

In Phase 1 (*introduction/in-scanner elicitation*), we fully explained the study; administered the initial questionnaires not relevant to the present report; and familiarized Lara with the MRI scanner and procedures. Then Lara entered the scanner, where we conducted a 10 min structural scan and a 5 min resting state scan according to standard fMRI research procedures (keep the eyes closed, stay relaxed and calm). Then we administered the imagination task, derived from a recent fMRI paradigm used by Belardinelli et al. (2009). Participants in the scanner were shown short written prompts to imagine saying (e.g., “to say ‘pencil’,” or “to say ‘lamp”’), seeing (“to see a pencil,” “to see a lamp”), hearing, feeling, or sensing something. The stimuli were presented in mini-blocks consisting of four prompts of one of the five categories, each shown for 7 s with 1 s inter-stimulus interval ( =32 s in total). Thus one mini-block consisted of four seeing prompts; another mini-block consisted of four saying prompts; and so on. Participants were instructed to imagine vividly what was shown on the screen for the duration of the presentation of the prompt. After four prompts a fixation cross was shown for 19 s before the next mini-block of prompts was presented.

In Phase 2 (*natural-environment DES*), we instructed Lara in the use of the DES beeper and the sampling task (Hurlburt and Heavey, 2006; Hurlburt, 2011): she was to wear the beeper in her natural environment for approximately 3 h, during which she would hear (through an earphone) six randomly occurring 700 Hz beeps. Her task was to terminate the beep (a button press) and then immediately to jot down notes about her ongoing inner experience that was “in flight” at the moment the beep sounded. Later that day or the next day she returned for a DES expositional interview about those six beeped experiences; this interview was conducted by RH and at least one and as many as four additional interviewers (the study was part of a training program), usually including some combination of SK, CF, and BAD. The expositional interviews were “iterative” (Hurlburt, 2009, 2011), designed to provide increasing-across-sampling-days skill in apprehending and describing inner experience. Following this interview, Lara returned to her everyday environment, during which (and on the same day) she responded to six more random beeps. The following day she returned for a second expositional interview about the second-sampling-day’s six beeped experiences. This sequence was repeated twice more, so that Lara sampled in four natural-environment periods, each followed by an expositional interview.

In Phase 3 (*in-scanner DES*), Lara (having been trained in DES in the natural environment) entered the scanner for a 25-min session with the instruction to keep the eyes open, stay relaxed and calm. That is, we did not set any particular task for her other than to respond to the DES beep (700 Hz, except these beeps were of 1.4 s duration); she had been instructed to note her experience that was ongoing *just prior* to the beep (that is, in the usual DES way). At four quasi-random times, she received a DES beep through a headphone. Immediately after each beep, she jotted a few notes about her experience on a clipboard positioned on her lap (viewable through a mirror); that is, this procedure mirrored as closely as possible the natural-environment DES procedure. Immediately after she exited the scanner she participated in a DES expositional interview (conducted by RH and some combination of SK and BAD) about each of her four randomly beeped experiences, in the order in which they appeared (although doubling back and looking ahead was allowed). This 25-min fMRI scan/four beeps with jotted notes/expositional interview sequence was repeated a total of nine times, resulting in 4 × 9 = 36 random samples of experience occurring in 25 × 9 = 225 min of fMRI scanning.

In Phase 4 (*post-DES resting state*), Lara entered the scanner for another 10 min structural scan and a 5 min standard resting-state scan. Immediately after exiting the scanner, she completed questionnaires not relevant here. Then she was candidly debriefed.

### SCANNING PROCEDURE

Images were collected on a 3T Magnetom Trio MRI scanner system (Siemens Medical Systems, Erlangen, Germany) using a 32-channel radio frequency head coil. Structural images were obtained using a three-dimensional T1-weighted magnetization-prepared gradient-echo sequence (MPRAGE) based on the ADNI protocol (www.adni-info.org) [repetition time (TR) = 2500 ms; echo time (TE) = 4.77 ms; TI = 1100 ms, acquisition matrix = 256 × 256 × 176, flip angle = 7; 1 mm × 1 mm × 1 mm voxel size]. Functional images were collected using a T2^∗^-weighted echo planar imaging (EPI) sequence sensitive to blood oxygen level dependent (BOLD) contrast (TR = 2000 ms, TE = 30 ms, image matrix = 64 × 64, FOV = 216 mm, flip angle = 80, voxel size 3 mm × 3 mm × 3 mm, 36 axial slices).

### fMRI DATA PRE-PROCESSING AND MAIN ANALYSIS

The fMRI data were analyzed using SPM8 software (Wellcome Department of Cognitive Neurology, London, UK). The first four volumes of all EPI series were excluded from the analysis to allow the magnetization to approach a dynamic equilibrium. Data processing started with slice time correction and realignment of the EPI datasets. A mean image for all EPI volumes was created, to which individual volumes were spatially realigned by means of rigid body transformations. The structural image was co-registered with the mean image of the EPI series. Then the structural image was normalized to the Montreal Neurological Institute (MNI) template, and the normalization parameters were applied to the EPI images to ensure an anatomically informed normalization. A commonly applied filter of 8 mm full-width at half maximum (FWHM) was used. Low-frequency drifts in the time domain were removed by modeling the time series for each voxel by a set of discrete cosine functions to which a cut-off of 128 s was applied. The statistical analyses were performed using the general linear model (GLM).

The imagination task was modeled as blocks with a duration of 32 s. The beeps of the DES procedure were modeled as events on the onset of the beep with a duration of 0. These vectors were convolved with a canonical hemodynamic response function (HRF) and its temporal derivatives.

For the DES procedure, we asked RH, on the basis of the DES expositional interviews he (and others) had conducted, to classify each of Lara’s 36 in-the-scanner experiences into four modalities: verbal, visual, bodily, and auditory (categories could overlap). We also asked RH to classify each of Lara’s experiences according to which (if any) of the 5FP (inner speaking, inner seeing, unsymbolized thinking, feeling, and sensory awareness) were present. RH’s classifications were checked by at least one other person who had been present at the relevant interview; disagreements were resolved by consensus. Then, regressors were built coding the categories that RH had assigned to the 36 events. For display purposes the resulting SPMs were thresholded at *p* < 0.001 and a significant effect was reported when the volume of the cluster was greater than the Monte-Carlo-simulation-determined minimum cluster size above which the probability of type I error was < 0.05 (AlphaSim; Ward, 2000). The resulting maps were overlaid onto a normalized T1 weighted MNI template (colin27) and the coordinates reported correspond to the MNI coordinate system.

## RESULTS

**FIGURE 1:**
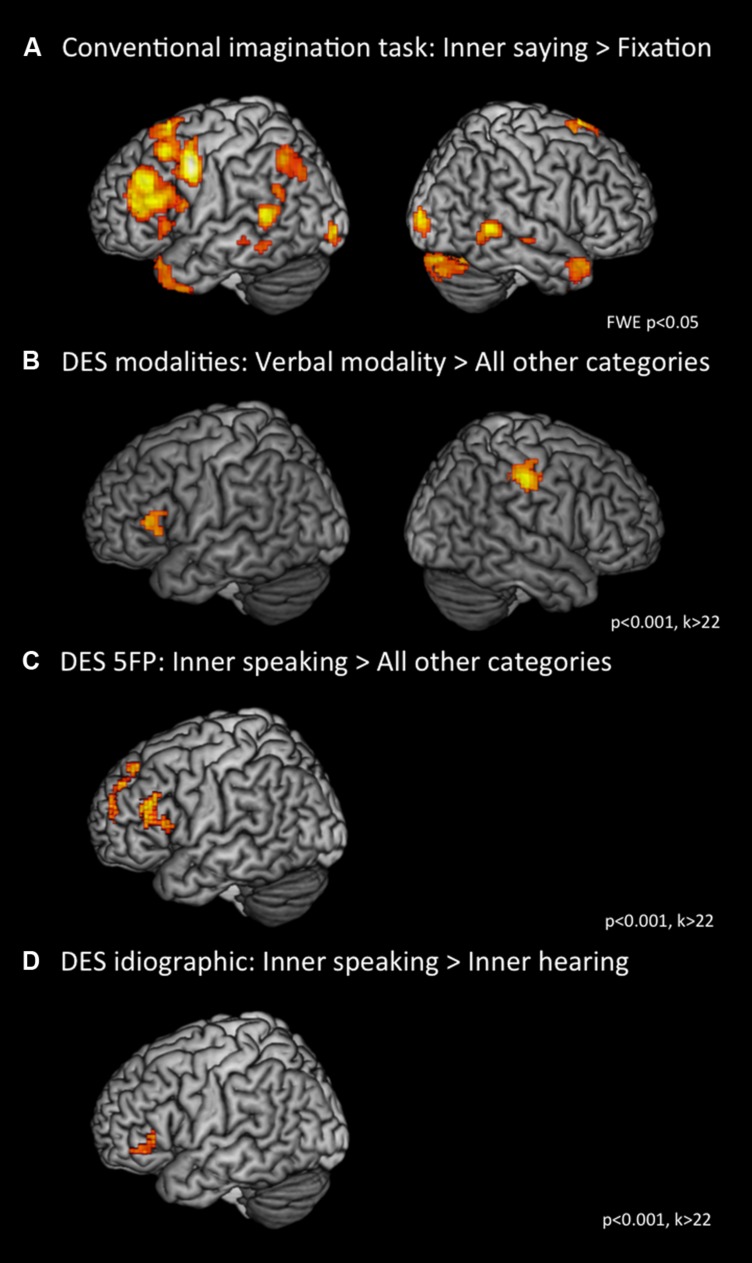
**(A)** Contrast of inner saying > fixation in a conventional neuroscientific mini-block design; **(B)** Contrast of verbal modality > all other modalities (visual, bodily, and auditory) as defined by DES; **(C)** Contrast of inner speaking > all other categories as defined by descriptive experience sampling (DES) 5 frequent phenomena (5FP); **(D)** Contrast of inner speaking > inner hearing of idiographic DES categories.

In Phase 1 (*introduction/in-scanner elicitation*) we investigated whether Lara would produce results that aligned with what conventional neuroscience would predict for inner speaking. **Figure 1A** shows a comparison of mini-blocks in which Lara had been instructed to imagine herself speaking (but not actually speaking aloud) compared against mini-blocks in which a fixation cross was shown. This figure shows that Lara’s brain produced the predicted activation of the inner speech network: left IFG and STS as well as superior and middle temporal gyrus (see also **Table 1A**).

**Table 1A:** Conventional imagination task: inner saying > fixation (FWE *p*< 0.05).

Area	BA	Peak coordinates (MNI)	*t*-score	Extent	*p*
Left rolandic operculum	6	-48, 2, 46	11.74	430	0.000
Left dorsolateral prefrontal cortex (DLPFC), inferior frontal gyrus (IFG)	46, 45	-39, 35, 34	9.14	337	0.000
Left middle temporal gyrus	21	-51, -49, 10	8.92	80	0.000
Left middle temporal gyrus	21	-66, -46, 4	8.06	77	0.000
Right visual cortex	18	30, -97, 4	8.00	78	0.000
Right cerebellum (Crus 2)		18, -82, -32	7.97	272	0.000
Left visual cortex	18	-33, -97, -5	7.62	23	0.000
Left angular gyrus	39	-45, -61, 49	7.02	107	0.000
Right temporal pole	38	51, 20, -29	6.60	103	0.000
Left temporal pole	38	-33, 20, -26	6.60	106	0.000
Ventromedial prefrontal cortex	11	-3, 53, -17	6.04	26	0.000
Left IFG	45	-51, 20, 1	5.98	25	0.000

There was no fMRI data collected during Phase 2. In Phase 3 we ask first whether the DES interviews conducted by RH are capable of classifying Lara’s verbal experience in ways that correspond to her neurophysiological activation. **Figure 1B** shows that when we compare Lara’s brain activity in those moments that RH classified as verbal to those classified as nonverbal (visual, bodily, or auditory), Lara showed the predicted activation of left IFG (see also **Table 1B**).

**Table 1B:** Descriptive experience sampling modalities: verbal modality > all other categories (*p*< 0.001, *k* > 22).

Area	BA	Peak coordinates (MNI)	*t*-score	Extent	*p*
Right postcentral gyrus	3	48, -22, 43	5.05	102	0.000
Left IFG	45	-51, 29, 10	4.24	31	0.000
Anterior cingulate gyrus	32	0, 20, 43	4.02	34	0.000

Next in Phase 3, we examine the 5FP category of inner speaking. Samples that included inner speaking were spread throughout the scanning sessions: three samples occurred during the third scanning session, one each during the fourth, seventh, and eighth scans, and two during the ninth scan. We modeled brain activity across all 36 samples as a function of whether RH had said inner speaking was or was not present. We did the same kind of univariate modeling across all 36 samples for each of the remaining four 5FP characteristics (that is, for inner seeing, for unsymbolized thinking, for feelings, and for sensory awareness). Then across the eight samples in which RH had said inner speaking was present, we compared the average of results of the inner speaking model to the average of the results of the four remaining models; this analysis indicated the presence of activity in left IFG, the core of the inner speech network (**Figure 1C**, **Table 1C**). We compared inner speaking to the non-inner-speaking samples instead of comparing against baseline because baseline includes times during which Lara was responding to (jotting down notes about) samples.

**Table 1C:** Descriptive experience sampling 5FP: inner speaking > all other categories (*p*< 0.001, *k* > 22).

Area	BA	Peak coordinates (MNI)	*t*-score	Extent	*p*
Left IFG	45	-36, 32, 19	4.96	88	0.000
Left DLPFC	9	-12, 41, 43	4.26	58	0.000

Because DES is primarily an idiographic technique, it is held to be capable of describing characteristics that apply to one individual, regardless of whether those characteristics are important for many or any other individuals. Therefore we asked RH to identify characteristics that might emerge from Lara’s participation in the DES procedure (during either or both the natural environment and the in-the-scanner phases) that are not standard modality features (verbal, visual, bodily, and auditory) and that are not identified as 5FP, regardless of whether the feature was a characteristic of any other DES participant. One such feature that RH noted was that when Lara experienced inner words, they ranged on a continuum from innerly spoken to innerly heard (Hurlburt et al., 2013). The distinction between speaking and hearing may be illustrated by the metaphor of speaking into a tape recorder (production) and hearing your voice being played back (reception). Contrasting Lara’s brain activity during moments of inner speaking vs. moments of inner hearing of her own voice resulted in increased activity in left IFG (**Figure 1D**, **Table 1D**).

**Table 1D:** Descriptive experience sampling idiographic: inner speaking > Inner hearing (*p*< 0.001, *k* > 22).

Area	BA	Peak coordinates (MNI)	*t*-score	Extent	*p*
Left IFG	45	-51, 41, -5	3.87	23	0.000


## DISCUSSION

To summarize, whether prompted using a conventional imagery-based fMRI paradigm or classified via use of the DES, Lara’s experiences of inner speaking were, as expected, reliably associated with activation in left IFG. This validates the suggestion that it is indeed possible for the DES procedure to apprehend features of inner experience and to do so as they naturally occur—the present study, for example, did not set out to investigate inner speaking; it set out to investigate naturally occurring characteristics of Lara’s inner experience *whatever those characteristics might be*. DES identified inner speaking as an important feature of Lara’s experience, and our study design allowed us then to demonstrate predicted fMRI activations during the occurrence of that feature.

The validation of high fidelity apprehensions of inner experience demonstrated in the present study should not be taken as a validation of all introspective or subjective reports—DES is, by Nisbett and Wilson’s (1977; cf. Hurlburt, 2011, p. 195) analysis, an exceptional method. Furthermore, the present study should not be taken as a validation of all DES-type reports—the demonstration here was by just one DES investigator (and his colleagues) with one participant. However, Hurlburt and Heavey (2002) showed that inter-rater reliability could be high between DES practitioners. A related question is the one raised previously, namely why we presented the case of Lara and not some or all of the other participants. First, it is not possible to combine disparate idiographic cases in a single journal article. Second, even if the case of Lara were the only interesting case of the five, it is enough to establish an important principle: fMRI data and particular-moment-experiential data can be profitably combined at least for some participants in some situations.

The combination of phenomenology and neurophysiology might be understood as a validation of DES: claims about private experience are always questionable, and the fact that the DES claims correlate with known neurophysiological results lends non-trivial support to the adequacy of the DES claims. However, the validation could be understood to operate the other way around: that the DES descriptions lend support to fMRI techniques as a way of investigating short-duration phenomenological characteristics. Either way, such a merger might answer important questions that are impossible even to pose without experiential data aimed at particular moments of consciousness. Here are two examples. First, Lara’s inner speaking results show that (at least for Lara) there is a phenomenological distinction between innerly speaking one’s voice and innerly hearing one’s voice being spoken. To our knowledge, such a distinction is typically not attended to in contemporary models of inner speech (Fernyhough, 2004; Scott, 2013), but it may be a crucial one for future studies. For example, theories of auditory verbal hallucinations (AVHs) that emphasize monitoring of inner speech (e.g., Frith, 1995) may benefit from investigation of localizable differences between inner speaking and inner hearing.

Also of interest was the relatively wider spread of activation associated with imagined inner speech (**Figure 1A**) as compared to unprompted moments of inner speaking classified by the DES (**Figure 1B,D**). It must be borne in mind that these activation maps have differing levels of temporal precision in that the imagery data come from a block design (continually producing bits of inner speech over an extended period of time), whereas the DES samples, by their nature, are targeted at very specific moments. Nevertheless, one could speculate that these results reflect genuine differences—that is, when Lara is prompted to imagine speaking, her *actual* inner speaking is somewhat different (possibly dramatically different) from her naturally occurring inner speaking both phenomenologically and neurologically (see Hurlburt et al., in preparation). One interpretation is that Lara’s actual experience following the inner speech prompt also included some processing of the prompt itself and some monitoring of inner speech production. It should be noted that prompted inner speech is an unnatural phenomenon (Jones and Fernyhough, 2007), occurring very rarely outside of psychological research laboratories. However, nearly all psychological studies of inner speech are either retrospective or of the prompted variety.

Given that this is a single case, we do not know whether distinctions such as between inner speaking and inner hearing and between prompted and unprompted inner speaking reflect idiosyncratic characteristics of Lara (and/or of RH) or are characteristic of other individuals who would regularly report inner speaking as part of their everyday experience. The present study cannot answer such questions, but it does provide a method whereby such questions might be answered.

Furthermore, this study does not by itself establish a principle about introspections in general, because it investigated only one method (DES) and one investigator (RH). For example, this study should *not* be understood as saying that we should simply believe people when they tell us they are talking to themselves; Hurlburt (2011) holds that people are often substantially mistaken about such reports unless an adequate method is used. This study should *not* be understood as saying that questionnaires about experience or non-DES experience sampling are valid descriptors of experience (Hurlburt and Heavey, 2014; Alderson-Day et al., in preparation; Hurlburt et al., in preparation). This study does not explore the boundaries or parameters of confidence in DES (or in RH)—that is, it does not characterize the situations where we can be more (or less) confident about the correspondence between self-reports and associated brain activity.

However, this study does suggest a new set of opportunities for cognitive neuroscience investigations. Most fMRI studies ask the participant in the scanner to perform a task that indirectly invokes a particular set of brain functions in the scanner. Whether receptive (e.g., merely viewing a flashing display) or active (e.g., memorizing syllables), the aim of those tasks is indirect in the sense that the task and stimuli are presumed to elicit the desired brain functions. That is, participants do not observe or report any aspect of their brain or mental function; they simply engage in the task that presumably indirectly evokes the brain function.

As noted in the Introduction, some fMRI studies ask participants in the scanner directly to rate their cognition or mental activity on some predefined measure (e.g., Christoff et al., 2009). But until now, no study has tried to link fMRI measurements to naturally occurring rather than task-elicited features of a participant’s experiential phenomena in the scanner (Hurlburt et al., in preparation). Now that we have established that such studies are possible, future investigations can explore the utility, limitations, and boundaries of such studies, for example comparing DES with other introspection methods and their correlation with brain activity.

## Conflict of Interest Statement

The authors declare that the research was conducted in the absence of any commercial or financial relationships that could be construed as a potential conflict of interest.
